# Disruptive technologies for hemodialysis: medium and high cutoff membranes. Is the future now?

**DOI:** 10.1590/21758239-JBN-2020-0273

**Published:** 2021-04-09

**Authors:** Thiago Reis, Siddiq Anwar, Francisco de Assis da Rocha Neves, Claudio Ronco

**Affiliations:** 1 Universidade de Brasília, Faculdade de Ciências da Saúde, Laboratório de Farmacologia Molecular, Brasília, DF, Brasil.; 2 San Bortolo Hospital, International Renal Research Institute of Vicenza, Department of Nephrology, Dialysis and Transplantation, Vicenza, Italy.; 3 Clínica de Doenças Renais de Brasília, Departamento de Nefrologia, Brasília, DF, Brasil.; 4 Academia Nacional de Medicina, Programa Jovens Lideranças Médicas, Rio de Janeiro, RJ, Brasil.; 5 Abu Dhabi Health Services (SEHA) Company, Department of Nephrology, Abu Dhabi, United Arab Emirates.; 6 University of Padova, Department of Medicine, Padova, Italy.

**Keywords:** Dialysis, Renal Dialysis, Uremia, Diálise, Diálise Renal, Uremia

## Abstract

In the past decade, a new class of hemodialysis (HD) membranes (high retention onset class) became available for clinical use. The high cutoff (HCO) and the medium cutoff (MCO) membranes have wider pores and more uniformity in pore size, allowing an increased clearance of uremic toxins. Owing to the mechanism of backfiltration/internal filtration, middle molecules are dragged by the convective forces, and no substitution solution is needed. The HCO dialyzer is applied in septic patients with acute kidney injury requiring continuous kidney replacement therapy. The immune response is modulated thanks to the removal of inflammatory mediators. Another current application for the HCO dialyzer is in hematology, for patients on HD secondary to myeloma-kidney, since free light chains are more efficiently removed with the HCO membrane, reducing their deleterious effect on the renal tubules. In its turn, the MCO dialyzer is used for maintenance HD patients. A myriad of clinical trials published in the last three years consistently demonstrates the ability of this membrane to remove uremic toxins more efficiently than the high-flux membrane, an evolutionary disruption in the HD standard of care. Safety concerns regarding albumin loss as well as blood contamination from pyrogens in the dialysate have been overcome. In this update article, we explore the rise of new dialysis membranes in the light of the scientific evidence that supports their use in clinical practice.

## Resumo

Na última década, uma nova classe de membranas de hemodiálise (HD) (classe de início de alta retenção) tornou-se disponível para uso clínico. As membranas de ponto de corte alto (HCO) e ponto de corte médio (MCO) têm poros mais largos e maior uniformidade no tamanho dos poros, permitindo uma maior depuração de toxinas urêmicas. Devido ao mecanismo de retrofiltração/filtração interna, as moléculas médias são arrastadas pelas forças convectivas, não sendo necessária uma solução de substituição. O dialisador de HCO é aplicado em pacientes sépticos com lesão renal aguda que requerem terapia renal substitutiva contínua. A resposta imunológica é modulada graças à remoção de mediadores inflamatórios. Outra aplicação atual para o dialisador de HCO é em hematologia, para pacientes em HD secundária ao rim do mieloma, uma vez que as cadeias leves livres são removidas mais eficientemente com a membrana de HCO, reduzindo seu efeito deletério sobre os túbulos renais. Por sua vez, o dialisador de MCO é utilizado para pacientes em HD de manutenção. Uma miríade de ensaios clínicos publicados nos últimos três anos demonstra consistentemente a capacidade desta membrana de remover toxinas urêmicas de forma mais eficiente do que a membrana de alto fluxo, uma ruptura evolutiva no padrão de cuidado em HD. As preocupações de segurança em relação à perda de albumina, bem como a contaminação do sangue por pirogênios no dialisato foram superadas. Neste artigo de atualização, exploramos o surgimento de novas membranas de diálise à luz das evidências científicas que apoiam seu uso na prática clínica.

## Introduction

A typical adult dialyzer has around 15,000 hollow fibers tightly packet, forming a compact bundle placed inside a 20 cm plastic tube. The sum of the inner area of these fibers varies usually from 1.4 to 2.1 m^2^, creating the blood-dialysate barrier, i.e., the membrane's surface area^1^^,^ 2. The fibers are made of synthetic polymers and the process of producing them in its essence is the same utilized in the textile industry to produce modern synthetic fabrics. Depending on controlled variables like temperature, pressure, percentage of materials in the blending, and exposure time in these conditions, unique membranes are manufactured. Recreating these same conditions over and over to produce membranes with the exact same properties at low cost in a mass-production fashion requires challenging logistics. The goal of bioengineers is to produce a biocompatible porous structure with optimized properties for water and solutes transport. Ideally, all the pores should have the same size to allow the adequate transport of uremic toxins^3^^-^ 6, while sparing the loss of essential proteins like albumin, soluble receptors, immunoglobulins, and proteins involved in the coagulation physiology. Characteristics such as pore size, uniformity in pore size, pore distribution, and density across the surface of the membrane will define its efficiency^7^.

Three physicochemical mechanisms define the clearance of solutes from the blood during hemodialysis (HD): diffusion, convection, and adsorption^8^. However, membranes with adsorptive properties are still in the pipeline and not commercially available for maintenance hemodialysis regimens^9^, only being currently used in the nephrointensivism setting^10^. Routinely employed filters for chronic HD defined as high-flux (HF) allow diffusive and convective clearances, having polyarylethersulfone (PAES) as its main compound^1^. The mean pore radius of these filters is 3.9 nm^11^^,^ 12 and they can be applied in conventional HD (high-flux HD [HF-HD]), or hemodiafiltration (high-flux hemodiafiltration [HF-HDF]). The HF-HD modality is limited in removing solutes with molecular weight greater than 15 kDa. In its turn, even utilizing the same membrane as in HF-HD, the online HF-HDF modality removes efficiently a broader range of molecules up to 25 kDa, thanks to high ultrafiltration volumes of around 100 mL/min, which represent the convective clearance. During a four-hour HF-HDF session, desirably 21 L or more of ultrafiltrate are generated. The minimum target volume of 21 L arouse from four major trials that demonstrated that long-term mortality reduction was achieved if at least 21 L of ultrafiltrate were generated in each HD session^13^. The difference between the volume of ultrafiltrate and the volume of reinfusion equals the net ultrafiltration volume. For example, if the ultrafiltrate volume is 21 L and the reinfusion volume 19 L, at the end of the session the patient will have a reduction of 2 kg (~2 L) in his body mass. An inherent consequence of high convective volumes is the increment in the transmembrane pressure^14^, associated with higher deposition of proteins in the inner surface of the hollow fibers, partial obstruction of pores, and loss of membrane efficiency during the treatment, a phenomenon termed as clogging^15^. In this update article we discuss the innovations in the field of HD membranes; Table 1 shows a list of core concepts and their definitions regarding HD membranes.

**Table 1:** Core concepts and their definitions regarding HD membranes

Concept	Definition
Sieving coefficient	The ratio between the solute concentration in the filtrate and the solute concentration in plasma water in the absence of a diffusion gradient across the membrane
Molecular weight retention onset	MW at which the sieving coefficient first reaches 0.9 (90%), the extraction from the blood of molecules with higher MW than the retention onset is 90% or less
Molecular weight cutoff	MW at which the sieving coefficient reaches 0.1 (10%), the extraction from the blood of molecules with higher MW than the cutoff is 10% or less
Reduction ratio	Subtraction of the pre-HD session concentration from the post-HD session concentration of a solute, divided by its pre-HD session concentration
Backfiltration/Internal filtration	Inflow of fluids across the dialysis membrane, from the dialysate compartment towards the blood compartment

HD, hemodialysis; MW, molecular weight.

### Disruptive technology

The technology employed for the development of medium cutoff (MCO) and high cutoff (HCO) dialyzers is disruptive since it provides equivalent or even higher removal of middle molecules in the conventional HD modality than HF-HDF modality without the need for a devoted hemodiafiltration (HDF) machine^16^^-^ 18 and the production of online reinfusion solution. Albeit using a similar blend of PAES and polyvinylpyrrolidone as utilized in HF filters, the mean pore radius for the MCO filter (5.0 nm) and HCO filter (10 nm) are higher than those of HF filters (3.9 nm)^11^^,^ 12.

In the late 1980s, the phenomenon described as backfiltration or internal filtration was discussed as a drawback of HD filters with enhanced permeability. Backfiltration refers to the switch on the fluid direction across the semipermeable membrane during the passage of blood through the hollow fibers. When the blood reaches the inlet of the dialyzer the resulting pressure gradient of oncotic and hydrostatic pressures in the blood compartment versus in the dialysate compartment dictate the extrusion of fluids from the blood. This pressure gradient is gradually reduced as the blood moves along the fibers and at a given point before reaching the outlet port the pressure gradient becomes negative, now favoring the movement of fluids from the dialysate compartment towards the blood compartment. Back in those days, ultrapure water technology was not available and microbial components such as endotoxins, peptidoglycans, and bacterial DNA eventually present in the dialysate fluid could gain access to the blood. Therefore, the backfiltration phenomenon had a negative connotation^19^^,^ 20. This perception radically changed as internal filtration was envisioned as a convective technique^21^ and an adjuvant mechanism for the removal of middle molecules.

### High Cutoff (HCO)

The potential role for the HCO membrane for maintenance hemodialysis patients was investigated in a randomized crossover trial in which 43 patients were divided to receive 3 weeks (9 sessions) of hemodialysis with either HCO or HF membranes and then switched to further 3 weeks with HCO if they started with HF or with HF for those that initially utilized the HCO filter^22^. A run-in phase of two weeks on HF-HD homogenized the groups and a washout period of two weeks was performed to reduce the carryover effect. The transcription rate of cytokine genes in peripheral blood leukocytes, related to a pro-inflammatory phenotype, was more discrete during the HCO phase. The transferrin receptor transcripts were higher in the HCO phase, a signal of improvement in erythropoiesis. The investigators selected pre-dialysis albumin (66 kDa) concentration as a safety endpoint since the molecular weight cutoff (MWCO) for the HCO membrane is 170 kDa^12^ (Table 2) and albumin removal is an undesirable effect of the HCO membrane. Indeed, the reduction in serum albumin was noticeable (from 36.2 ± 3.5 to 31.0 ± 4.7 g/L, P <0.01). For this reason, the implementation of chronic HD regimens using the HCO dialyzer was deemed unsafe and discouraged. Of note, a recent randomized trial that compared 49 patients divided to receive 12 weeks of either MCO-HD (medium cutoff hemodialysis) or HF-HD demonstrated that the MCO-HD group required a reduced dose of erythropoiesis-stimulating agents^23^, clinically corroborating the molecular findings previously reported^22^.

**Table 2:** Characteristics of dialyzers

Dialyzer	Type	Purpose	Mean pore radius	Inner diameter/wall thickness	Company	MWRO	MWCO	Availability in Brazil
EMiC2®	HCO	CKRT	10 nm	220 µm/35 µm	Fresenius*	15 kDa	170 kDa	YES
SepteX®	HCO	CKRT	10 nm	215 µm/50 µm	Gambro/Baxter§	15 kDa	170 kDa	NO
Theralite®	HCO	HD dysproteinemias	10 nm	215 µm/50 µm	Gambro/Baxter§	15 kDa	170 kDa	NO
Theranova®	MCO	HD	5 nm	180 µm/35 µm	Gambro/Baxter§	9 kDa	56 kDa	YES

CKRT, continuous kidney replacement therapy; HCO, high cutoff; HD, hemodialysis; MCO, medium cutoff; MWCO, molecular weight cutoff; MWRO, molecular weight retention onset.

* Fresenius Medical Care GmbH, Bad Homburg, Germany.

§ Gambro Dialysatoren GmbH, Hechingen, Germany. Baxter Healthcare Corporation, Deerfield, IL, USA.

Another potential use for the HCO membrane was explored in patients with acute kidney injury stage 3D (requiring HD) secondary to biopsy-proven light chain cast nephropathy. The molecular weight of kappa and the lambda free light chains (FLC) is 23 and 45 kDa, respectively. The MYRE trial randomized 98 patients with cast nephropathy to carry out HD with either HCO or HF membranes. The primary outcome was dialysis independence at 3 months, being similar in both groups. At 6 and 12 months, dialysis independence was higher in the HCO group. The reduction ratio (RR) (Table 1), for kappa and lambda was higher in the HCO group, confirming its ability to remove more efficiently FLC. The EuLITE trial had a similar design and randomized 90 patients. Again, no difference in dialysis independence at 3 months was found^24^. The authors hypothesized that bortezomib-based chemotherapy alone is highly effective for early reductions in FLC, potentially blunting beneficial effects of the mechanical removal of FLC by the HCO dialyzer.

A well-established use for the HCO membrane is in continuous kidney replacement therapy (CKRT), precisely for continuous veno-venous hemodialysis (CVVHD). The membranes usually employed for CKRT are HF, applying convective modalities, i.e., continuous veno-venous hemodiafiltration (CVVHDF) and continuous veno-venous hemofiltration (CVVH). Both modalities are attractive options since they promote higher clearance of middle molecules such as myoglobin (17 kDa) than CVVHD with an HF membrane. The drawback of convective modalities is related to elevated filtration fraction and elevated transmembrane pressure^14^ that are inherent to these procedures, being associated with a reduction in filter life span^25^. Weidhase et al^26^. proved in a randomized trial with 60 individuals that the clearances of ß2-microglobulin (12 kDa), myoglobin (17 kDa), and interleukin 6 (26 kDa) were higher in the group that carried out CVVHD with HCO membrane versus the group that carried out CVVHD with HF membrane. Importantly, there was no difference in albumin losses. This apparent incongruence with the detectable losses reported in chronic HD patients might be related to blood flow influence on albumin removal. In CVVHD, blood flow is around 120 mL/min, whereas in chronic HD is 400 mL/min. As backfiltration increases proportionally with blood flow^2^, the clearance of middle and large molecules such as albumin follows the same pattern.

### Medium cutoff (mco)

**Figure 1:**
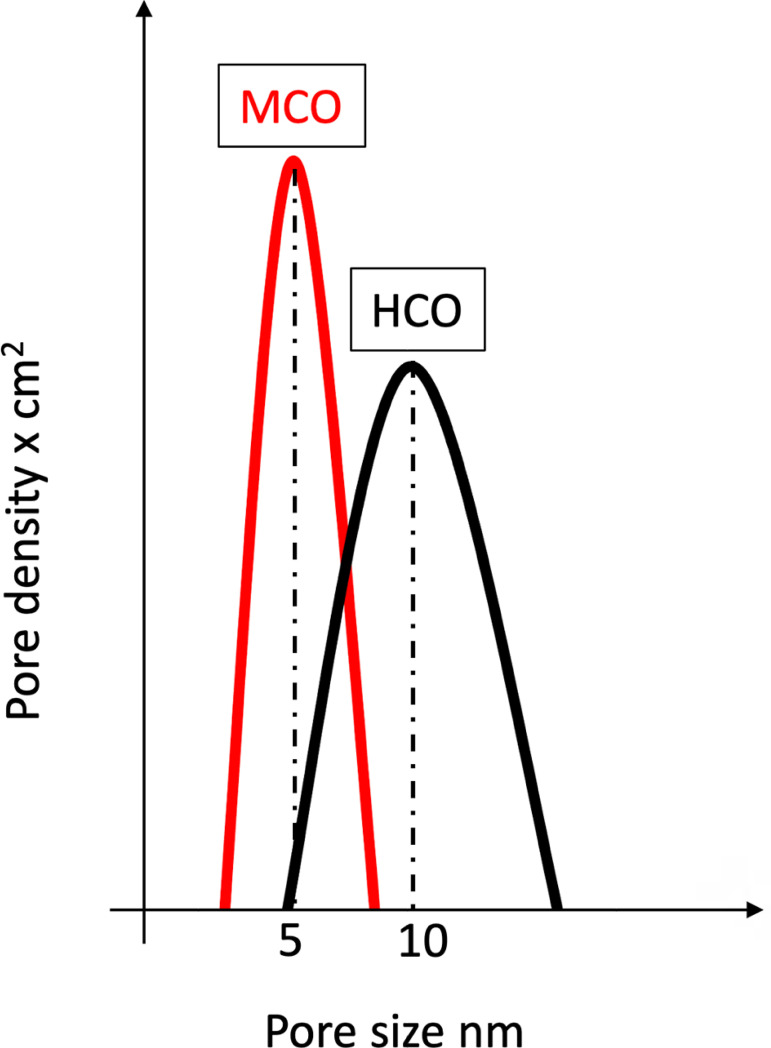
Pore size distribution of medium cutoff (MCO) and high cutoff (HCO) membranes. The MCO membrane mean pore radius is 5 nm, standard deviation 0.1 nm. The HCO membrane mean pore radius is 10 nm, standard deviation 2.0 nm.

Ideally, all the pores of a membrane should have the same size and tridimensional configuration. However, this degree of perfection cannot be achieved. For any HD membrane, pores of different sizes are scattered in a Gaussian distribution (Figure 1). The evolutionary leap from the HCO to the MCO technology resides in a narrower range of distribution regarding pore size. The mean pore radius is 5 nm, standard deviation 0.1 nm, for the MCO membrane, with a lower variance from the mean, i.e., more uniformity in pore dimension^27^, providing a more selective removal of solutes with reduced albumin leakage. As a comparison, the HCO membrane mean pore radius is 10 nm with a wider standard deviation (2.0 nm)^28^. Kirsch et al. observed an albumin loss of 3 g in a four-hour MCO-HD session, which is similar to the loss seen in HF-HDF^17^. Besides, the MCO fiber's inner diameter (180 µm) is 17% thinner than in HCO (215 µm), a feature that enhances the backfiltration mechanism^2^, detailed by our group 20 years ago^29^. The concern about albumin leakage has been extensively explored. It is noteworthy that in clinical studies that applied the membrane for three months or less, albumin pre-dialysis concentration was indeed reduced when compared to baseline after the intervention^30^^-^ 34. This finding was challenged by two multicenter trials; one was a single-arm study that applied the MCO membrane for six months in patients (n=87) previously on HF-HD regimen^35^. The other study was a randomized controlled trial comparing one group of individuals on MCO-HD (n=65) versus another group on HF-HD (n=65), also for six months^36^. Altogether, 152 patients carried out MCO-HD for six months and pre-dialysis serum albumin remained stable. We speculate that in a time point between three to six months a catch-up phenomenon takes place and pushes albumin concentration back to baseline. This paradoxical behavior still remains to be elucidated and it might be influenced by a reduction in the pro-inflammatory phenotype of the patients, owed by the increased removal of uremic toxins in MCO-HD.

**Figure 2:**
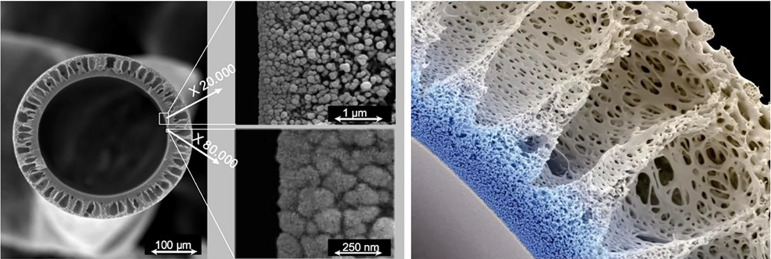
Structural characteristics of the medium cutoff dialyzer. Scanning electron microscopy images of the fiber (left), internal skin layer (middle), and fiber wall (right).

A naturally posed question about safety in membranes with enhanced internal filtration is the risk of inadvertent inflow of bacterial degradation products from the dialysate towards the blood compartment in case of water system contamination. An in vitro elegant model compared the permeability of a low-flux, HF, MCO, and HCO membranes, when exposed to a contaminated dialysate with filtrates of two water-borne bacteria and endotoxin concentration four-fold higher than the maximum allowed^11^. The results demonstrated a similar concentration of endotoxins in the blood compartment, irrespective of the membrane. This was a proof of concept study, demonstrating that the permeability of the MCO and HCO membranes to middle molecules favors a one-way direction since the external layer of these membranes and the tridimensional configuration repeal middle molecules and only allow the entrance of small molecules dragged in by the backfiltration mechanism (Figure 2). Another similar study in design yielded similar results. Specifically for the *Pseudomonas aeruginosa* extract added to contaminate the dialysate, MCO and HCO membranes were less permeable for endotoxins than low-flux and HF membranes, providing a protective profile^28^.

In the head-to-head comparison of HF-HD versus MCO-HD, the RR of middle molecules such as ß2-microglobulin (12 kDa), cystatin C (13 kDa), myoglobin (17 kDa), prolactin (23 kDa), kappa FLC (23 kDa), complement factor D (24 kDa), α1-acid glycoprotein (41 kDa), lambda FLC (45 kDa) was robustly higher in MCO-HD^17^^,^ 31 ^,^ 36 ^,^ 37. A step further is the comparison of the RR in HF-HDF versus MCO-HD. The bulk of data shows at least non-inferiority between these two modalities especially regarding soluble solutes with a molecular weight above 15 kDa, such as myoglobin, prolactin, complement factor D^17^^,^ 18 ^,^ 38. Of note, a higher RR does not necessarily imply a sustained reduction in the pre-dialysis concentration of a given uremic toxin. Belmouaz et al. demonstrated that myoglobin and prolactin have higher RR in MCO-HD versus HF-HD, albeit pre-dialysis concentration did not reduce^31^. These findings illustrate the complexity involved in the kinetics of middle molecules concerning their concentration in different body compartments and the magnitude of the rebound effect after a dialysis session. The REMOVAL-HD trial^35^ showed not only a higher RR but also a sustained reduction in the pre-dialysis concentration of FLC favoring MCO-HD over HF-HD. It is highly debatable if the better clinical outcomes achieved with HF-HDF^13^ can be extrapolated for MCO-HD, based on the fact that the removal of middle molecules is similar for both modalities. Finally, the possibility of utilizing the MCO filter for CKRT seems appealing as it merges the advantages of CVVHD (increased filter life span) and CVVH/CVVHDF (higher clearance of inflammatory mediators)^39^.

## Conclusion

It seems that the endeavor to mimic the glomerular filtration barrier has reached its climax with MCO and HCO membranes. Exploring the mechanism of backfiltration/internal filtration resembles a miniaturization of the hardware and disposables deployed in HF-HDF. In a way, a dreamed future about HD membranes has become a reality. However, other key kidney functions executed by the renal tubules, such as secretion and absorption of solutes, are far from being replicated by current membrane technology. The nephrology community should be familiarized with the history of membrane evolution, and the developments in the past 40 years should not be taken for granted. The interaction between nephrologists and bioengineers to fulfill unmatched patient needs will point the direction for new breakthrough discoveries in the field of renal replacement therapy.

## Abbreviations

HD: hemodialysis

HF: high-flux

PEAS: polyarylethersulfone

HF-HD: high-flux hemodialysis

HF-HDF: high-flux hemodiafiltration

MCO: medium cutoff

HCO: high cutoff

HDF: hemodiafiltration

MWCO: molecular weight cutoff

MCO-HD: medium cutoff hemodialysis

FLC: free light chain

RR: reduction ratio

CKRT: continuous kidney replacement therapy

CVVHD: continuous veno-venous hemodialysis
